# Modeling *Culicoides paraensis* distribution and implications for Oropouche virus transmission in Brazil

**DOI:** 10.1186/s13071-025-07144-9

**Published:** 2025-11-27

**Authors:** Camila Lorenz, Thiago Salomão de Azevedo, Alekin Bispo Gomes, Francisco Chiaravalloti-Neto, Maria Anice Mureb Sallum

**Affiliations:** 1 Department of Parasitology, Instituto Butantan, São Paulo, Sao Paulo Brazil; 2 Department of Epidemiology, School of Public Health of University of Sao Paulo, São Paulo, Brazil; 3 Secretary of Health, Municipality of Santa Barbara d’Oeste, Sao Paulo, Brazil; 4 Department of Biodiversity, Institute of Biosciences, UNESP, Rio Claro, Sao Paulo Brazil

## Abstract

**Abstract:**

Oropouche virus (OROV) is mainly transmitted to humans by *Culicoides paraensis*, a biting midge widely distributed across the Americas. In this study, we modeled the potential distribution of *C. paraensis* in Brazil using environmental variables and found that temperature-related factors, particularly minimum temperature and annual temperature range, were the strongest predictors of its occurrence. Comparison of the predicted distribution with confirmed autochthonous OROV cases revealed several areas of mismatch, suggesting either underreporting of *C. paraensis* or the involvement of additional vector species in transmission. These findings highlight the need to integrate *C. paraensis* into Brazil’s arbovirus surveillance systems and to strengthen entomological monitoring with the support of remote sensing, climate data, and ecological research to better anticipate and mitigate future transmission risks.

**Graphical Abstract:**

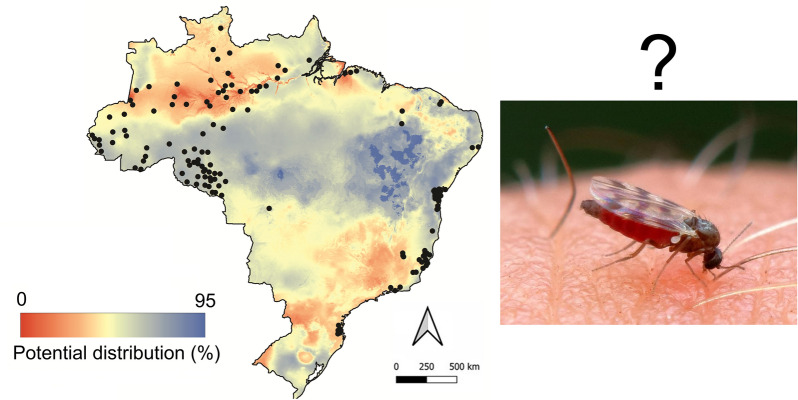

The Oropouche virus (OROV) is not a novel pathogen. First isolated in 1955, this arthropod-borne Orthobunyavirus has long been associated with sporadic outbreaks in tropical forested areas of South America, particularly within the Brazilian Amazon [1]. However, since late 2023, OROV has expanded its geographic range, with transmission reported in the Caribbean and in previously non-endemic areas of Brazil and Central America, likely driven by the emergence of recombinant viral lineages [2]. In 2024, a total of 16,239 confirmed cases of Oropouche fever were reported in the Region of the Americas [3]. Of these, 7236 autochthonous cases were molecularly confirmed in Brazil, representing a sharp increase compared to the previous year and highlighting both the expanding geographic range and the growing public health significance of OROV transmission [3]. Alarmingly, fatal outcomes in adults have been documented for the first time [4].

OROV is primarily transmitted to humans by *Culicoides paraensis*, a biting midge broadly distributed throughout the Americas, from the US to Argentina. This vector is well adapted to anthropogenic environments and displays opportunistic feeding behaviour, targeting both humans and domestic animals, factors that facilitate its role in urban and peri-urban transmission cycles [5].

We conducted an extensive review of the scientific literature to identify confirmed occurrences of *C. paraensis* across Brazil. Using occurrence records from 1960 to 2025, combined with environmental variables, we developed a potential distribution model employing the Maxent machine learning algorithm [6]. The Maxent algorithm is a machine learning method widely used to model species distributions based on presence-only data [6]. It estimates the probability distribution of a species across geographic space by finding the distribution of maximum entropy, subject to environmental constraints derived from known occurrence records. In practice, Maxent compares environmental conditions at presence locations with background data across the study area, identifying the most informative variables that explain the species’ ecological niche. The output is a continuous map of habitat suitability, indicating areas with higher or lower likelihood of species presence. Due to its robustness with limited data and strong predictive performance, Maxent has become one of the most frequently applied tools in ecological niche modeling and biodiversity studies.

A total of 170 records of *C. paraensis* were identified in Brazil between 1960 and 2025. The variables most strongly associated with its distribution were: (1) minimum temperature of the coldest month, (2) annual temperature range, (3) precipitation seasonality, and (4) precipitation of the driest month. The ecological niche model for *C. paraensis* demonstrated robust predictive performance (AUC = 0.823 for training; 0.738 for testing), well above random expectation, confirming strong discriminatory power between suitable and unsuitable habitats. Temperature-related factors were the primary determinants of species distribution, accounting for ~ 70% of model contribution. The minimum temperature of the coldest month was the most influential variable (45.5%), underscoring the species’ sensitivity to extreme cold as a major ecological constraint. The annual temperature range (24.3%) further highlighted the role of thermal stability, with limited tolerance to seasonal variability restricting distribution to more climatically stable regions. Precipitation variables, though less influential (30% combined), were still significant, with precipitation seasonality (17%) outweighing precipitation of the driest month (13.2%). These results suggest that both thermal thresholds and rainfall patterns shape the species’ potential range, particularly by limiting persistence in colder or highly seasonal environments.

The model indicated the highest probabilities of species presence in humid tropical forests and floodplain areas of northern Brazil and the western Amazon basin, particularly in the states of Pará, Amazonas, Acre, and Rondônia, as well as in parts of the Central-West and Southeast regions (Fig. 1). In contrast, regions characterized by tropical savanna and seasonally dry tropical forest exhibited low environmental suitability for this vector. However, when comparing the predicted potential distribution of *C. paraensis* with the locations of confirmed autochthonous Oropouche fever cases in Brazil [7], we identified several areas of non-overlap (Fig. 1). To evaluate spatial autocorrelation between areas susceptible to *C. paraensis* and the occurrence of Oropouche cases, we generated local indicator of spatial association (LISA) cluster maps for 2020 and 2024 (Fig. 2). These maps allowed us to identify local spatial patterns between the two variables and highlight critical areas where Oropouche fever is more likely to occur in association with the presence of *C. paraensis*.

**Fig. 1:**
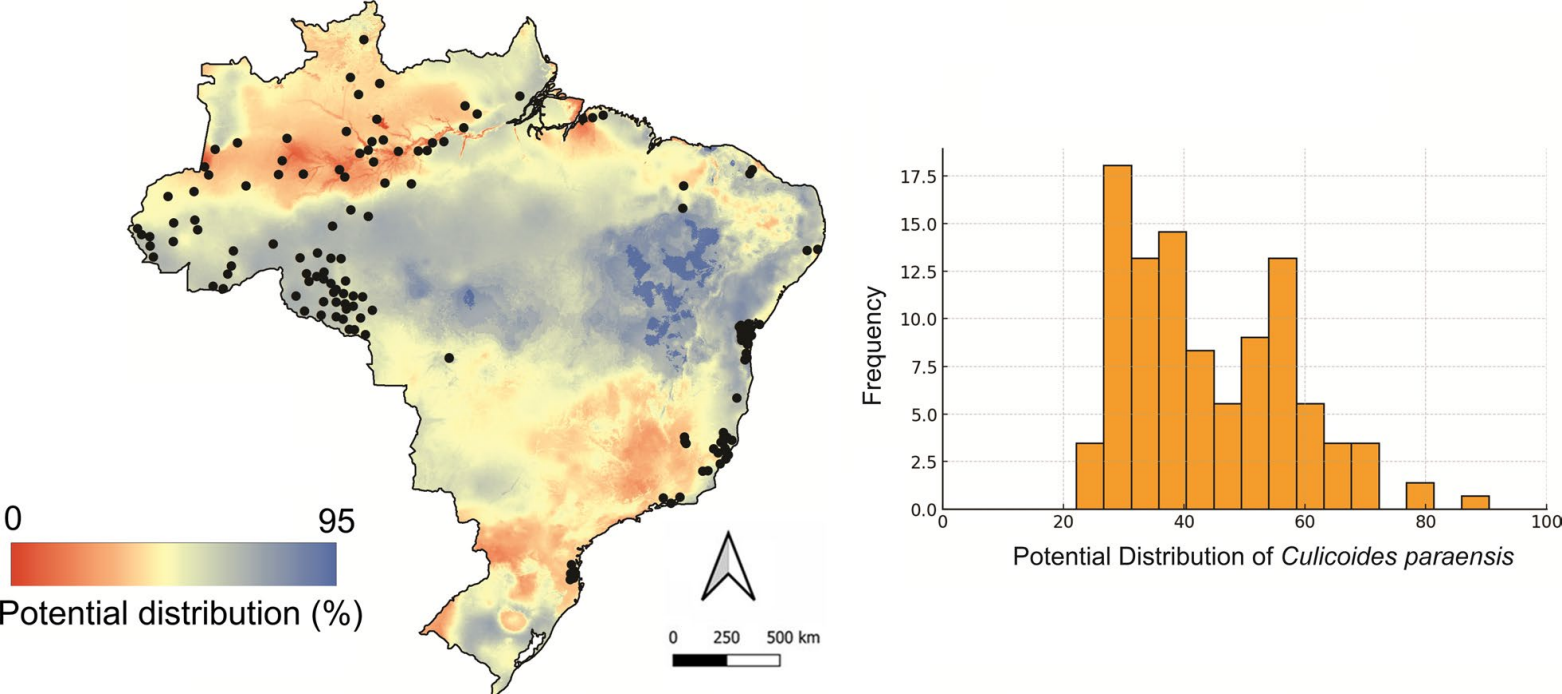
*Culicoides paraensis* potential distribution in Brazil. Left: The red areas indicate regions with the highest predicted probability of *C. paraensis* occurrence, based on ecological niche modeling using Maxent and distribution records reported in the literature. Black dots represent autochthonous ORO cases reported in 2024. Right: Frequency of municipalities with autochthonous ORO cases according to the environmental suitability for *C. paraensis*. While the vast majority of cases occurred in areas with high predicted suitability for the vector, a few cases were identified in regions with low probability of *C. paraensis* presence—underscoring the need for improved entomological data and surveillance in these areas

**Fig. 2:**
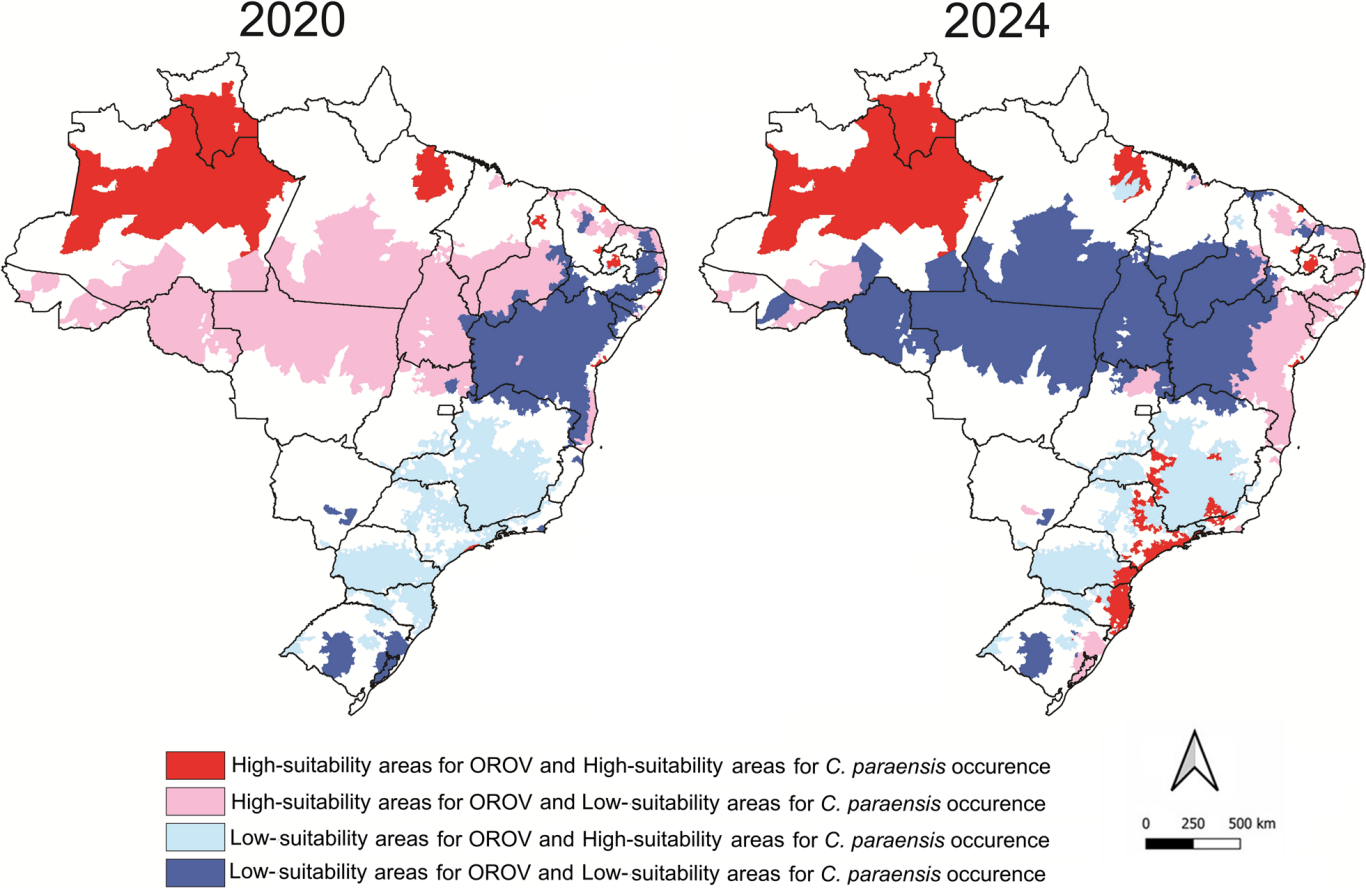
Local indicator of spatial association (LISA) cluster maps for occurrence of OROV cases and *Culicoides paraensis*

The most immediate and plausible explanation for this mismatch is that *C. paraensis* may be present in these regions but has not yet been formally reported in epidemiological bulletins or scientific literature. Alternatively, other vector species may also be contributing to OROV transmission in these non-overlapping areas. A systematic review found that in the 60 years spanning from the identification of Oropouche virus to the 2023–2024 epidemic in the Americas, only seven studies investigating vector competence had been published [5]. *Culicoides sonorensis*, another species of biting midge, displayed a high competence for OROV transmission, with infection rates around 30%. Mosquitoes (Diptera: Culicidae) are believed to play a secondary role in the transmission of OROV. Species such as *Coquillettidia venezuelensis* and *Aedes serratus* have been proposed as potential sylvatic vectors, while *Culex quinquefasciatus* has been suggested as a candidate for urban transmission [1]. However, available evidence indicates that infection rates in both *Aedes* and *Culex* species consistently remain < 20%, with limited capacity to support OROV transmission [5]. It is also possible that genetic changes to the virus have improved vector competence, similar to what has been seen with chikungunya virus and *Aedes albopictus* [8] or West Nile virus and *Culex* spp. mosquitoes [9]. This possibility is particularly relevant for certain coastal areas of South Bahia and Espírito Santo, major cocoa-producing regions that have reported a substantial number of autochthonous OROV fever cases occurring outside the endemic Amazon region, despite a scarcity of entomological surveys or confirmed records of the primary vector*, C. paraensis* in these areas. This highlights a critical gap in surveillance and reinforces the urgent need for more sensitive and targeted entomological monitoring, particularly in agroecosystems where *Culicoides* larvae thrive in microhabitats rich in decaying organic matter, such as plant debris from banana and cocoa plantations [10].

Past studies on the distribution of *C. paraensis* in Brazil have been limited by small sample sizes, narrow geographic scope, and inconsistent methodologies, which reduce statistical power, introduce spatial bias, and constrain the environmental conditions represented in models. Therefore, predictive maps often lack reliability and generalizability, undermining their value for surveillance and public health planning. To address these gaps, future research should expand spatial and temporal sampling, standardize collection methods, and apply advanced analytical approaches—such as occupancy or hierarchical Bayesian models—to account for imperfect detection and uncertainty. Coordinated, large-scale efforts are essential to generate robust, actionable insights into the ecology and public health risks associated with *C. paraensis.*

In light of the findings, integrating *C. paraensis* into Brazil’s arbovirus monitoring frameworks is essential for strengthening preparedness against Oropouche virus (OROV). Current strategies focus almost exclusively on *Aedes aegypti*, leaving biting midges overlooked despite their key epidemiological role. Expanding research is urgently needed to better characterize the distribution of *C. paraensis* across Brazil and the wider Americas while also assessing the vector competence of other insect species that may contribute to OROV transmission. In the context of climate change, marked by rising temperatures and shifting precipitation patterns, the geographic range of known vectors may expand, and previously non-competent species could acquire transmission capacity. Strengthening entomological surveillance, supported by remote sensing and climate data, and investing in vector ecology research are therefore critical to anticipating future risks, guiding targeted control in vulnerable communities, and ensuring OROV prevention strategies are aligned with One Health and health equity principles.

## Data Availability

All data used in this analysis are available in https://www.gov.br/saude/pt-br/assuntos/saude-de-a-a-z/o/oropouche/painel-epidemiologico.
